# Efficacy of Multi-Component Exercise-Based Injury Prevention Programs on Injury Risk Among Footballers of All Age Groups: A Systematic Review and Meta-analysis

**DOI:** 10.1007/s40279-022-01797-7

**Published:** 2023-02-08

**Authors:** Rilind Obërtinca, Ilir Hoxha, Rina Meha, Arber Lama, Altina Bimbashi, Dorentina Kuqi, Bujar Shabani, Tim Meyer, Karen aus der Fünten

**Affiliations:** 1 Institute of Sports and Preventive Medicine, Saarland University, Saarbrücken Campus, Building B8 2, 66123 Saarbrücken, Germany; 2 Dartmouth Institute for Health Policy and Clinical Practice, Lebanon, NH USA; 3 Department of Physiotherapy, University of Gjakova “Fehmi Agani”, Gjakova, Kosovo; 4 Research Unit, Heimerer College, Pristina, Kosovo; 5 Evidence Synthesis Group, Pristina, Kosovo; 6 University Clinical Center of Kosovo, Pristina, Kosovo

## Abstract

**Background:**

Playing football is associated with a high risk of injury. Injury prevention is a priority as injuries not only negatively impact health but also potentially performance. Various multi-component exercise-based injury prevention programs for football players have been examined in studies.

**Objective:**

We aimed to investigate the efficacy of multi-component exercise-based injury prevention programs among footballers of all age groups in comparison to a control group.

**Methods:**

We conducted a systematic review and meta-analysis of randomized and cluster-randomized controlled trials. CINAHL, Cochrane, PubMed, Scopus, and Web of Science databases were searched from inception to June 2022. The following inclusion criteria were used for studies to determine their eligibility: they (1) include football (soccer) players; (2) investigate the preventive effect of multi-component exercise-based injury prevention programs in football; (3) contain original data from a randomized or cluster-randomized trial; and (4) investigate football injuries as the outcome. The risk of bias and quality of evidence were assessed using the Cochrane Risk of Bias Tool and the Grading of Recommendations Assessment, Development, and Evaluation (GRADE), respectively. The outcome measures were the risk ratio (RR) between the intervention and the control group for the overall number of injuries and body region-specific, contact, and non-contact injuries sustained during the study period in training and match play.

**Results:**

Fifteen randomized and cluster-randomized controlled trials with 22,177 players, 5080 injuries, and 1,587,327 exposure hours fulfilled the inclusion criteria and reported the required outcome measures. The point estimate (RR) for the overall number of injuries was 0.71 (95% confidence interval [CI] 0.59–0.85; 95% prediction interval [PI] 0.38–1.32) with very low-quality evidence. The point estimate (RR) for lower limb injuries was 0.82 (95% CI 0.71–0.94; 95% PI 0.58–1.15) with moderate-quality evidence; for hip/groin injuries, the RR was 0.56 (95% CI 0.30–1.05; 95% PI 0.00–102.92) with low-quality evidence; for knee injuries, the RR was 0.69 (95% CI 0.52–0.90; 95% PI 0.31–1.50) with low-quality evidence; for ankle injuries, the RR was 0.73 (95% CI 0.55–0.96; 95% PI 0.36–1.46) with moderate-quality evidence; and for hamstring injuries, the RR was 0.83 (95% CI 0.50–1.37) with low-quality evidence. The point estimate (RR) for contact injuries was 0.70 (95% CI 0.56–0.88; 95% PI 0.40–1.24) with moderate-quality evidence, while for non-contact injuries, the RR was 0.78 (95% CI 0.55–1.10; 95% PI 0.25–2.47) with low-quality evidence.

**Conclusions:**

This systematic review and meta-analysis indicated that the treatment effect associated with the use of multi-component exercise-based injury prevention programs in football is uncertain and inconclusive. In addition, the majority of the results are based on low-quality evidence. Therefore, future high-quality trials are needed to provide more reliable evidence.

**Clinical Trial Registration:**

PROSPERO CRD42020221772.

**Supplementary Information:**

The online version contains supplementary material available at 10.1007/s40279-022-01797-7.

## Key Points

**Table:** 

The present meta-analysis is the first to use prediction intervals in the interpretation of results derived from trials assessing the efficacy of multi-component exercise-based injury prevention programs among footballers of all age groups.
This study revealed that the evidence for meaningful effects of exercise-based injury prevention programs remains inconclusive at best.
The quality of evidence is a major issue in existing studies; therefore, these findings call for future high-quality trials to provide more reliable evidence.

## Background

The overall injury incidence in professional male football players is between 5.9 [1] and 9.6 [2] injuries/1000 football hours. In amateur and veteran football, reported incidences are even higher and reach 9.6 [2] to 12.5 [3] and 12.4 [4] injuries/1000 football hours, respectively. There are hardly any data regarding players under the age of 11 years [5]. A professional football team with 25 players has approximately 50 injuries per season [6], and youth elite teams about 30 [7]. Many efforts have been made in recent years to reduce these numbers. Various injury prevention programs for football players of both sexes and various age groups have been established. Some of them target specific injuries, for example, Prevent injury and Enhance Performance [8] and HarmoKnee [9], target knee injuries. Others take a more general approach, trying to prevent non-contact lower extremity injuries in general for example, FIFA^®^ 11 [10], FIFA^®^ 11 + [11], and the Neuromuscular training program [12]. 11 + Kids [13] aims to prevent football injuries by increasing children’s fundamental and sport-specific motor skills.

Previous systematic reviews and meta-analyses have evaluated the efficacy of either specific programs (e.g., FIFA 11 and 11 +) [14, 15] or the effect of various programs on specific injuries (e.g., non-contact injuries) [16]. However, recognizing the differences between programs regarding the content, the different age groups targeted, and the different results reported compared to each other, a comprehensive meta-analysis of pooled results across the studies will produce a more comprehensive result. To date, no meta-analysis is available that has evaluated the efficacy of all multi-component exercise-based injury prevention programs in reducing the overall number of injuries as well as body region-specific injuries, and considering footballers of all age groups (children, youth, senior, and veteran). Additionally, contact-related injuries represent 50% of overall injuries in professional football [17]. Previous research has not investigated the impact of the programs on preventing these injuries. Providing information about the age-specific efficacy and estimating the potential of these programs on contact-related injuries may guide future evidence-based directions regarding the implementation and development of new interventions. Finally, providing only confidence intervals (CIs) might not be the best way forward. A recent meta-analysis examined the effect of the Nordic hamstring exercise [18]. The authors strongly recommended providing the prediction intervals (PIs) in addition to CIs. This is in line with authors promoting the use of PIs in the interpretation of results from a random-effects meta-analysis of trials assessing treatment effects [19]. Therefore, and for the first time, this meta-analysis reports the PIs in addition to the CIs. The aim of this meta-analysis was to investigate the efficacy of multi-component exercise-based injury prevention programs in reducing injuries of different types among footballers of all age groups.

## Methods

### Protocol and Registration

We report this systematic review in accordance with the guidelines of the Preferred Reporting Items for Systematic reviews and Meta-Analyses (PRISMA) [20]. The study was registered at PROSPERO (ID: CRD42020221772).

### Study Eligibility Criteria

In the present study, we included all controlled, multi-component exercise-based injury prevention programs containing at least two or more exercises. Players of the intervention group performed these programs during their training sessions in addition to their usual training and were compared to a control group. Criteria for study inclusion were: (1) include football (soccer) players; (2) investigate the preventive effect of multi-component exercise-based injury prevention programs in football; (3) contain original data from a randomized or cluster-randomized trial; and (4) investigate football injuries as the outcome. Studies were excluded from the meta-analysis if they were: (1) studies with a single exercise intervention; (2) studies with a primary target on performance or other physical measurements than injuries; (3) studies using protective equipment (e.g., bracing) as part of the intervention; and (4) studies published in a language other than English.

### Sources and Study Selection

Possible studies were identified using a systematic search process. First, we searched the following databases CINAHL, Cochrane, PubMed, Scopus, and Web of Science from the earliest record to June 2022, with the following search strategy: (injury prevention OR warm-up program OR neuromuscular program OR f-marc OR 11 +) AND (football OR soccer). The reference lists of the studies recovered were hand searched to identify potentially eligible studies missed by electronic searches. Two reviewers independently (AB, DK) performed the selection of studies based on the title and abstract provided by the bibliographic databases. The full-text evaluation followed on those selected studies from the first selection step. A third reviewer (RO) was responsible for resolving any discrepancies in the selection process.

### Data Extraction and Administration

For each eligible study, four reviewers (RM, AB, DK, AL) extracted data independently using a standardized data extraction form [14]. One section was added (type of injuries: contact or non-contact) to the extraction form for an additional analysis that we performed regarding the effect on contact versus non-contact injuries. We extracted data on the studies’ basic information, design, participants, intervention characteristics, and outcome measures. Thereafter, the reviewers compared the extracted data for consistency. Reviewers resolved discrepancies by discussion and, when necessary, a fifth party (RO) was involved. Final decisions were made based on a majority vote. Primary outcome results from individual studies were extracted and collated in Excel 365 (Microsoft Corporation, Redmond, WA, USA).

### Quality Assessment

The risk of bias was assessed for each included trial according to the recommendations outlined in the Cochrane Handbook for Systematic Reviews of Interventions [21]. The following items were considered: allocation sequence generation, concealment of allocation, blinding of outcome assessment, incomplete outcome data, selective outcome reporting, and other sources of bias. As it is impossible to blind the participants to the intervention, we removed the item “blinding of participants and investigators”. Each bias domain was judged as at low or high risk of bias according to its possible effect on the results of the study. When the possible effect was unknown or insufficient detail was reported, we judged it as unclear. The risk of bias was examined independently by two reviewers (RO, BSH). Discrepancies were resolved by consensus. The overall quality of evidence was assessed using the Grading of Recommendations Assessment, Development, and Evaluation (GRADE). This method assesses the strength of evidence derived from systematic reviews [22]. In the GRADE system, randomized controlled trials (RCTs) begin as high-quality evidence [23]. Subsequently, the evidence is downgraded by one level for each of the following domains considered: (1) risk of bias (downgraded by one level if the trials scored an overall high risk of bias on the Cochrane Collaboration Risk of Bias Tool); (2) inconsistency (downgraded by one level if statistical heterogeneity between studies was *I*^2^ > 50%); (3) indirectness (downgraded by one level if the meta-analysis included participants with heterogeneous characteristics with regard to sex, age, and level of sport); (4) imprecision (downgraded by one level if the upper and lower CIs had a > 0.5 difference); and (5) publication bias (assessed with a visual inspection of a funnel plot and two-tailed Egger’s test if more than ten studies were included in the meta-analysis). Evidence obtained was categorized into four levels of evidence quality: high, moderate, low, and very low [24] (Table 1).

**Table 1:**
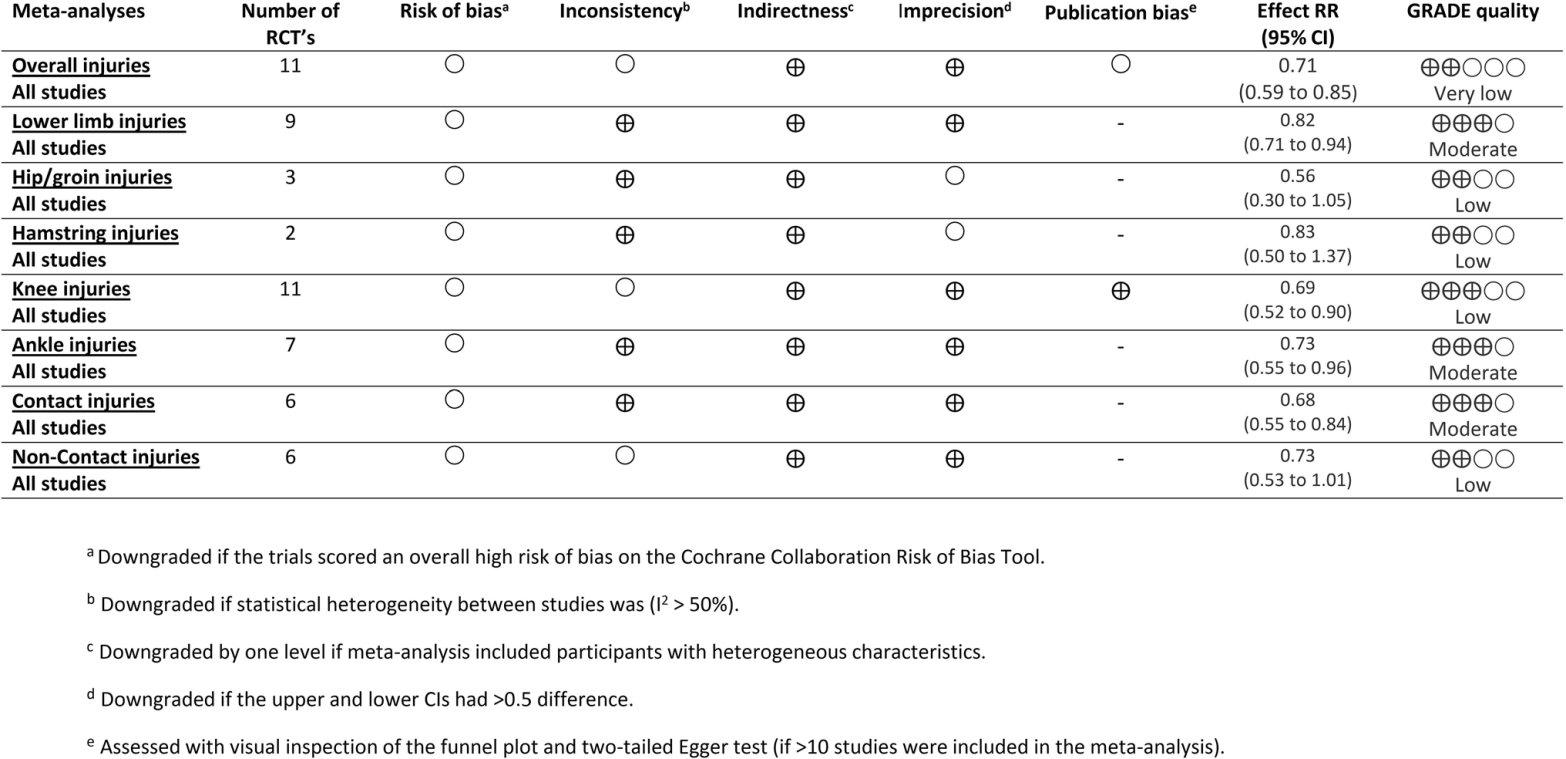
Grades of recommendation, assessment, development and evaluation (GRADE) quality of evidence

*CI* confidence interval, *RCT* randomized controlled trial, *RR* risk ratio

### Outcome Measures

The primary outcome was the risk ratio (RR) for the overall number of injuries. Body region-specific injury RRs for the lower limb, hamstring, hip/groin, knee, and ankle were secondary outcomes. Additionally, the overall number and the region-specific injury RRs were assessed for a non-contact versus contact induced cause. All injuries occurring in official training and match play during the respective study period were included.

### Synthesis of Results

If studies did not report RR estimates, we converted them to RRs as far as possible [25, 26]. Out of the 15 included studies, six studies did not perform cluster adjustments. They also did not provide information on the intra-cluster correlation coefficient or other data that would allow for calculating the design effect or inflation factor (as recommended by the Cochrane Handbook for Systematic Review of Interventions) [27]. Hence, we performed a cluster adjustment by increasing variance by 30% for effect estimates of studies with no adjustment for the cluster effect [28]. We performed a meta-analysis of RRs and their 95% CIs using the DerSimonian and Laird random-effects method [29]. A random-effects meta-analysis assumes that the true treatment effect varies among studies. The DerSimonian and Laird method does not make any assumptions about the distribution of the random effects [30]. In addition to the presentation of overall effect estimates and 95% CIs, we also calculated 95% PIs. They enable the examination of treatment effects within an individual study setting, as this can differ from the average effect [19]. Heterogeneity was assessed using *I*^2^, *τ*^2^, and *Q* value (*χ*^2^ test for heterogeneity). We interpreted *I*^2^ values according to guidelines by Higgins and Green, a low heterogeneity for *I*^2^ values between 25 and 50%, a moderate *f* heterogeneity or 50–75%, and a high heterogeneity for ≥ 75% [27]. A small study effect was investigated using Egger’s test for a meta-analysis with ten or more studies [31]. Statistical analysis was carried out using STATA 17 BE (Stata Corporation, College Station, TX, USA).

## Results

### Literature Identification

**Fig. 1:**
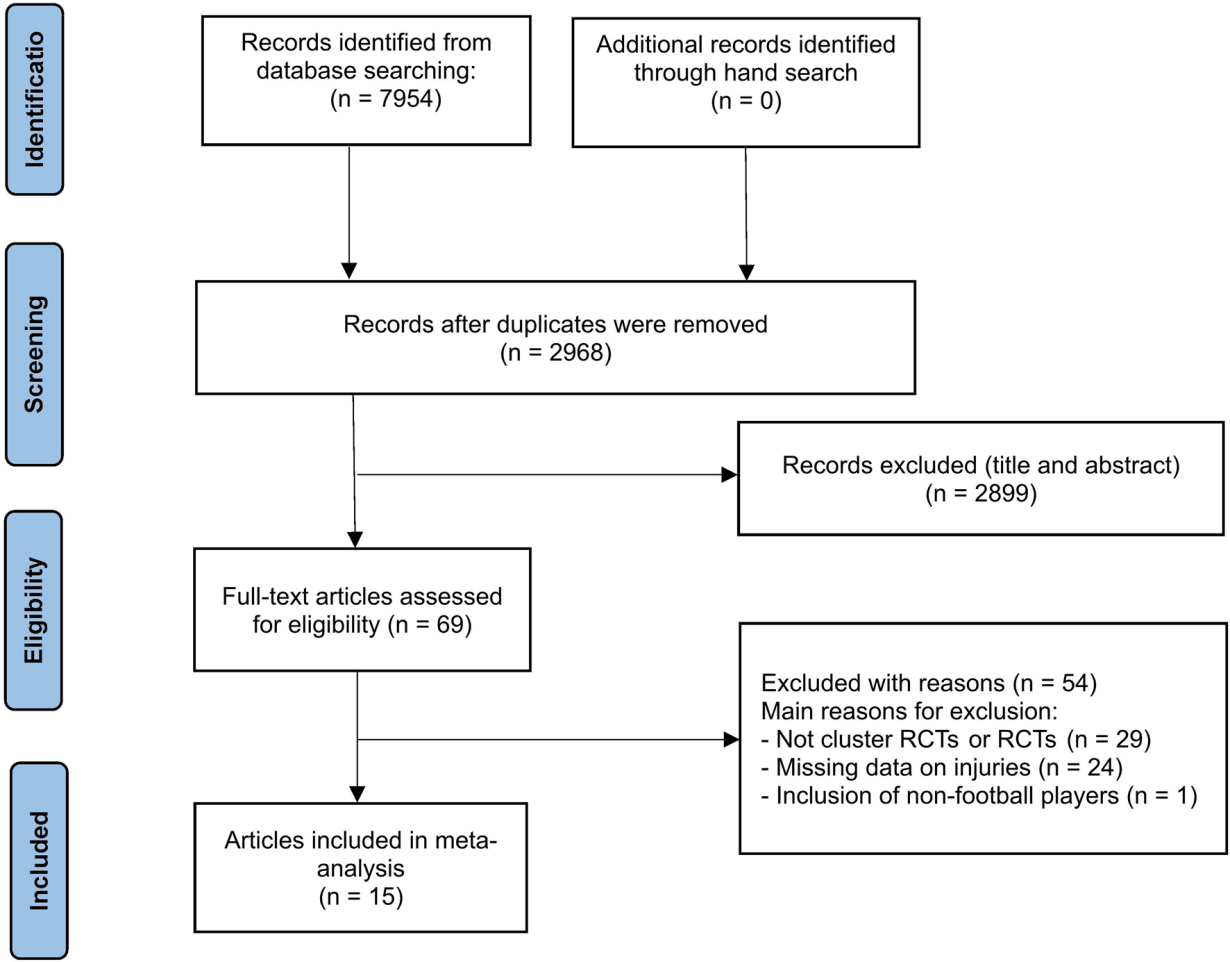
Flow chart of the included studies. *RCTs* randomized controlled trials

The initial database search identified 7954 studies. Following the removal of duplicates (*n* = 4986), 2968 studies remained. After screening the titles and abstracts, 69 full-text articles were left. A further 54 studies had to be excluded as they did not present data on injuries, included non-football players, or were neither cluster RCTs nor RCTs. Finally, 15 articles were included in the meta-analysis (Fig. 1).

### Demographic and Study Characteristics

Eight trials stemmed from Europe [4, 9–11, 13, 32–34]. Two trials were conducted in the USA [8, 35]. One trial was conducted in one each of the following countries: Canada [12], Australia [36], Rwanda [37], Nigeria [38], and Iran [39]. The overall number of participants was 22,177 including both sexes. Participants were registered football players in one of the following age groups: children (7–14 years), youth (12–19 years), senior, and veteran (> 32 years). The number of participants ranged from 265 [4] to 4564 participants [9]. A total of 5080 injuries and 1,587,327 h of exposure were included. The study period lasted between 12 weeks [8] and 9 months [4, 13, 39, 33]. All interventions were applied at least twice a week in the training sessions. The control groups performed their usual warm-up exercises and/or training routines. One study required an additional home-based stretching program [12]. Nine studies used a FIFA^®^ warm-up program of the FIFA^®^ 11, the FIFA^®^ 11 + , or the 11 + Kids [4, 10, 11, 13, 33, 35, 37–39]. Two studies used Neuromuscular Training programs [12, 32], and one study each used the Neuromuscular Control Program [36], the Knäkontroll program [9], the Prevention Injury and Enhance Performance program [8], and the Bounding Exercise Program [34] (Table 2).

**Table 2:** Summary of included multi-component randomized controlled trials investigating the effect of injury prevention programs

Study	Intervention program	Population (age)	Follow-up	Outcome	Number of analysed (players)	Exposure time (h)	Number of injuries
Emery et al. 2010 [12]	Neuromuscular training program	Male and female youth (13–18 years**)**	20 weeks	Overall injuries	IG: 380 CG: 364	IG: 24 051 CG: 24 597	IG: 50 CG: 79
Finch et al. 2016 [36]	Neuromuscular control program	Male senior (18–30 years)	28 weeks	Overall injuries	IG: 679 CG: 885	IG: 12 790^a^ CG: 15 537^a^	IG: 335 CG: 438
Gilchrist et al. 2008 [8]	PEP	Female senior, (19.88 years)^b^	12 weeks	Knee injuries	IG: 583 CG: 852	IG: 35 220 CG: 52 919	IG: 40^c^ CG: 58^c^
Hammes et al. 2015 [4]	FIFA^®^ 11 +	Male veteran (≥ 32 years)	9 months	Overall injuries	IG: 146 CG:119	IG: 4 172 CG: 2 937	IG: 51 CG: 37
Hilska et al. 2021 [32]	Neuromuscular training	Male and female children (9–14 years**)**	20 weeks	Lower limb injuries	IG: 673 CG: 730	IG: 71 109 CG: 63 404	IG: 310^d^ CG: 346^d^
Nuhu et al. 2021 [37]	FIFA^®^ 11 +	Male senior (IG: 19.9 years) (CG: 19.7 years))	7 months	Overall injuries	IG: 309 CG: 317	IG: 65 333 CG: 63 389	IG: 168 CG: 252
Owoeye et al. 2014 [38]	FIFA^®^ 11 +	Male youth (14–19 years)	6 months	Overall injuries	IG: 212 CG: 204	IG: 51 017 CG: 61 045	IG: 36 CG: 94
Rossler et al. 2018 [13]	11 + Kids	Male and female children (7–13 years**)**	9 months	Overall injuries	IG: 2066 CG: 1829	IG: 140 716 CG: 152 033	IG: 139 CG: 235
Silvers-Granell et al. 2017 [35]	FIFA^®^ 11 +	Male senior (18–25 years)	5 months	Overall injuries	IG: 675 CG: 850	IG: 35 226 CG: 44 212	IG: 285 CG: 665
Soligard et al. 2008 [11]	FIFA^®^ 11 +	Female youth (13–17 years)	8 months	Overall injuries	IG: 1055 CG: 837	IG: 49 899 CG: 45 428	IG: 161 CG: 215
Steffen et al. 2008 [10]	FIFA^®^ program 11	Female youth (13–17 years)	8 months	Overall injuries	IG: 1073 CG: 947	IG: 66 423 CG: 65 725	IG: 242 CG: 241
Walden et al. 2012 [9]	Knakontrol	Female youth (12–17 years)	7 months	ACL injuries	IG: 2479 CG: 2085	IG: 149 214 CG: 129 084	IG: 7^e^ CG: 14^e^
Zarei et al. 2020 [39]	11 + kids	Male children (7–14 years**)**	9 months	Overall injuries	IG: 443 CG: 519	IG: 31 934 CG: 32 113	IG: 30 CG: 60
Van de Beijsterveldt et al. 2012 [33]	FIFA^®^ program 11	Male senior (18–40 years)	9 months	Overall injuries	IG: 233 CG: 233	IG: 21 605 CG: 22 647	IG: 207 CG: 220
Van de Hoef et al. 2019 [34]	BEP	Male senior (18–45 years)	39 weeks	Hamstring injuries	IG: 229 CG: 171	IG: 31 831 CG: 21 717	IG: 35^f^ CG: 30^f^

*ACL* anterior cruciate ligament, *BEP* bounding exercise program, *CG* control group, *IG* intervention group, *N/A* Not applicable, *PEP* Prevent injury and Enhance Performance

^a^Match exposure only was reported

^b^Average age only was reported

^c^Knee injuries

^d^Lower limb injuries

^e^ACL injuries

^f^Hamstring injuries

### Risk of Bias

Seven (46%) studies had a high risk of bias in two or more domains. The domain “other bias” was the most frequent cause for a high risk of bias within the studies (46%), with seven studies neither reporting an intention-to-treat analysis nor an adjustment for clustering (Fig. 1 and Table 1 of the Electronic Supplementary Material [ESM]).

### Meta-Analysis Results

#### Overall, Body Region, Contact, and Non-Contact-Related Injuries

**Fig. 2:**
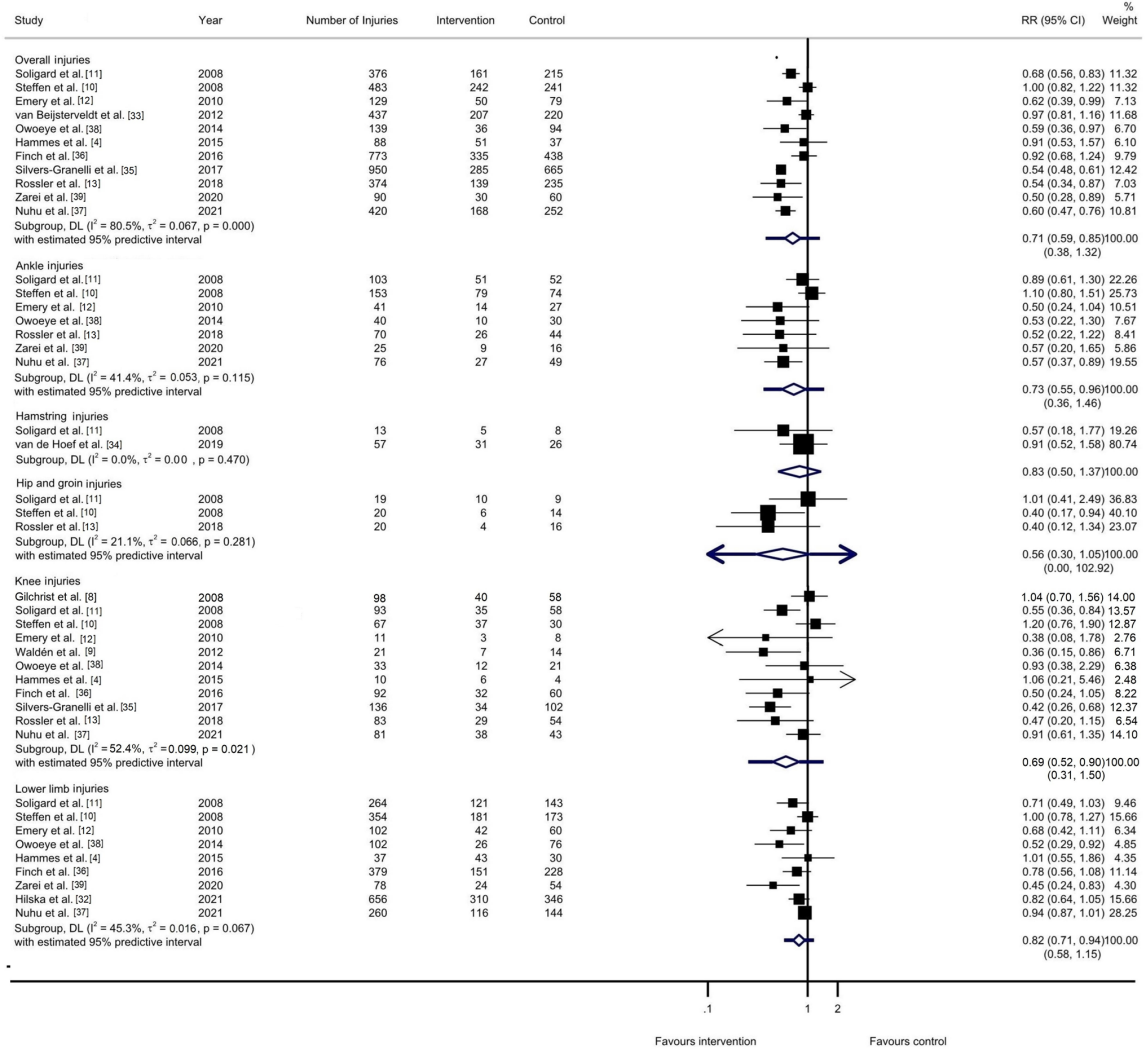
Analysis of multi-component exercise-based injury prevention programs’ effect on the overall and region-specific injury risk compared with control groups. *I*^*2*^ *I* square, *p* *p* value, *RR* risk ratio, *τ*^*2*^ tau square

For the primary outcome analysis, i.e., the overall injury risk, the pooled results showed a point estimate (RR) of 0.71 (95% CI 0.59–0.85; 95% PI 0.38–1.32; *I*^2^ = 80.5%; *τ*^2^ = 0.067; *p* < 0.001). The width of the 95% PI suggests that the effect in future similar studies lies between 0.38 and 1.32 (Fig. 2). In practical terms, the effect may vary from being very protective to an increased risk of injury. The level of evidence was rated as very low (downgraded one level because of a risk of bias, one level because of inconsistency, and one level because of publication bias) (Table 1).

Regarding the secondary outcome analyses, i.e., the body region-specific injury risk (Fig. 2), the point estimate (RR) for the lower limb injuries was 0.82 (95% CI 0.71–0.94; 95% PI 0.58–1.15; *I*^2^ = 45.3%; *τ*^2^ = 0.016; *p* = 0.067) with moderate-level evidence (downgraded one level because of a risk of bias). For knee injuries, the RR was 0.69 (95% CI 0.52–0.90; 95% PI 0.31–1.50) with low-level evidence (downgraded one level because of a risk of bias and one level because of inconsistency). For hip/groin injuries, the RR was 0.56 (95% CI 0.30–1.05; 95% PI 0.00–102.92) with low-level evidence (downgraded one level because of a risk of bias and one level because of imprecision). For hamstring injuries, the RR was 0.83 (95% CI 0.50–1.37) with low-level evidence (downgraded one level because of a risk of bias and one level because of imprecision). With regard to ankle injuries, the RR was 0.73 (95% CI 0.55–0.96; 95% PI 0.36–1.46) with moderate-level evidence (downgraded one level because of a risk of bias). For each calculation, the 95% PI was wider in comparison to the 95% CI.

**Fig. 3:**
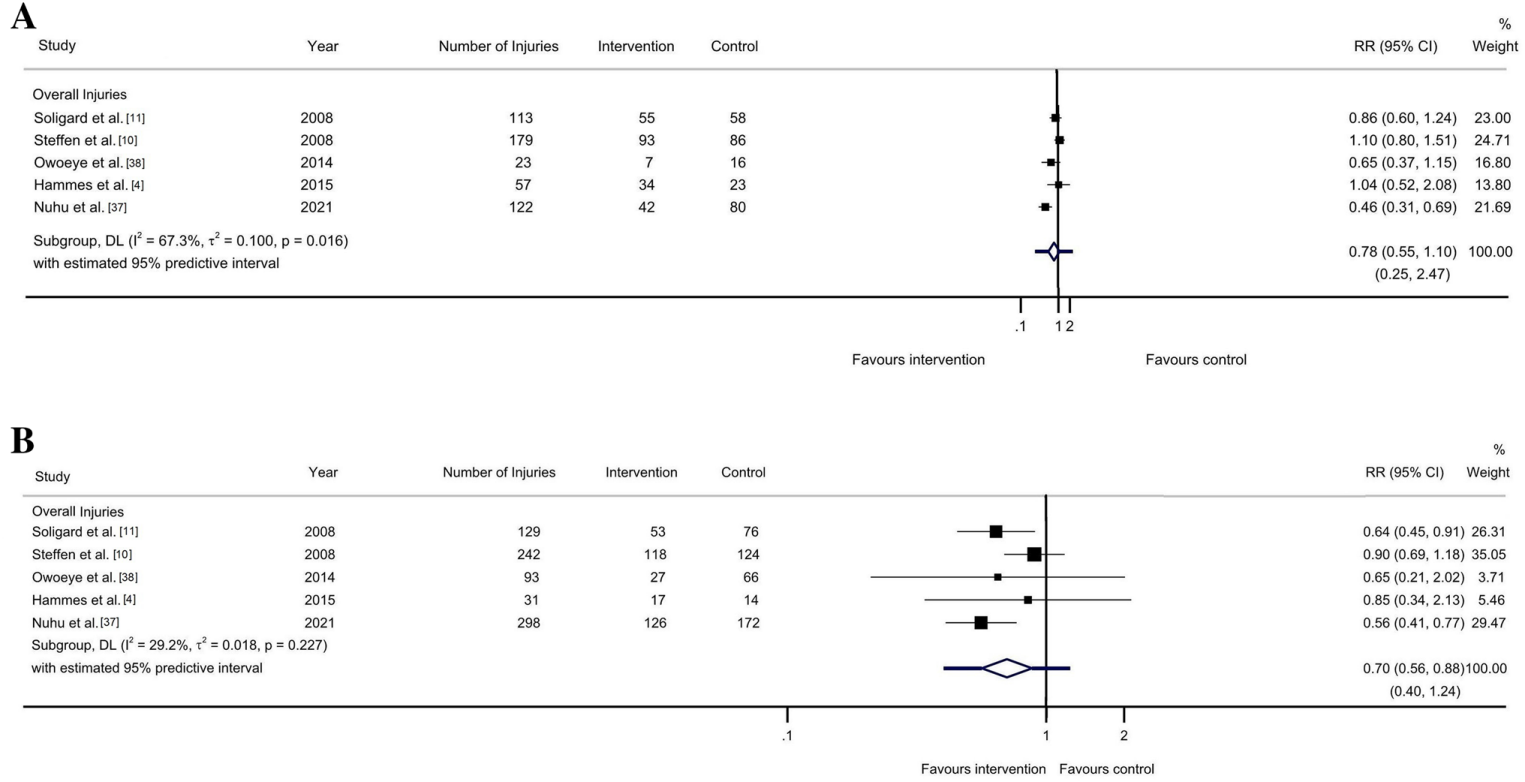
Analysis of multi-component exercise-based injury prevention programs’ effect on the overall non-contact (**a**) and contact (**b**) injury risk compared with control groups. *I*^*2*^ I square, *p* *p *value, *RR* risk ratio, *τ*^*2*^ tau square

The pooled results for non-contact injuries showed a point estimate (RR) of 0.78 (95% CI 0.55–1.10; 95% PI 0.25–2.47; *I*^2^ = 67.3%; *τ*^2^ = 0.100; *p* = 0.016), with evidence rated as low level (downgraded one level because of a risk of bias and one level because of inconsistency). Additionally, the point estimate (RR) for contact injuries was 0.70 (95% CI 0.56–0.88; 95% PI 0.40–1.24 *I*^2^ = 29.2%; *τ*^2^ = 0.018; *p* = 0.227), with moderate-level evidence (downgraded one level because of a risk of bias). The width of the 95% PI suggested that the effect may vary from being very protective to an increased risk of injury for both outcomes, i.e., non-contact injuries (95% PI 0.55–1.10) and contact injuries (95% PI 0.40–1.24) (Fig. 3).

#### Subgroup Analysis According to Sex

Regarding a distinction between male and female individuals, the point estimate (RR) for the overall number of injuries in male football players was 0.70 (95% CI 0.55–0.90; *I*^2^ = 83.5%; *τ*^2^ = 0.082; *p* < 001). In female football players, the point estimate (RR) was 0.82 (95% CI 0.57–1.20; *I*^2^ = 68.9%; *τ*^2^ = 0.064; *p* = 0.008) (Fig. 4 of the ESM).

#### Subgroup Analysis According to Age Group

The point estimate (RR) for the overall number of injuries in children was 0.52 (95% CI 0.36–0.76; *I*^2^ = 0.0%; *τ*^2^ < 0.001; *p* = 0.841), in youth, the RR was 0.74 (95% CI 0.56–0.97; *I*^2^ = 68.9%; *τ*^2^ = 0.048; *p* = 0.022), in seniors, the RR was 0.73 (95% CI 0.53–1.01; *I*^2^ = 91.1%; *τ*^2^ = 0.098; *p* < 0.001), and, in veterans, the RR was 0.91 (95% CI 0.53–1.57) (Fig. 4 of the ESM).

## Discussion

### Principal Findings

This systematic review and meta-analysis included 15 RCTs that assessed the effect of injury prevention programs on the overall and body region-specific injury risk in football players. Based on calculated PIs, their efficacy remains uncertain and inconclusive regarding all primary and secondary outcomes. In addition, the majority of the results are based on low-quality evidence.

#### Comparison with Existing Literature on Injury Risk Reduction

Riley et al. [40] suggested that if a random-effects approach is used, the pooled result must be interpreted as the average intervention effect across studies, rather than the common effect. Previous meta-analyses have not reported PIs, which means, an appropriate comparison is not possible. Therefore, we can only compare our point estimates with those reported in the literature. In contrast with the currently available evidence [14–16, 41], our study included footballers of all age groups and skill levels (amateur and professional). The point estimate (RR) of 0.71 (95% CI 0.59–0.85) in the current analysis is at the lower end of those reported in previous systematic reviews, which reported an incidence rate ratio (IRR) of 0.73 (95% CI 0.59–0.91) [41], IRR of 0.75 (95% CI 0.57–0.98) [14], IRR of 0.77 (95% CI 0.64–0.91) [15], and IRR of 0.77 (95% CI 0.61–0.97) [16]. This was to be expected as we also included interventions in children, which showed a substantially higher injury reduction of 48% [13] and 50% [39] compared with older players. This effect was somewhat counterbalanced by the reduced effect of the programs among veterans, which was only 9%. However, the relative weight of the studies with children was higher (higher in the number of studies and participants). A previous meta-analysis [14] investigated the effect of the FIFA^®^ exercise-based injury prevention programs on specific body regions. The observed efficacy on hamstring (RR 0.83 vs IRR 0.40), knee (RR 0.69 vs IRR 0.52), and ankle injuries (RR 0.73 vs IRR 0.68) was lower in our study, but comparable for hip/groin injuries (RR 0.56 vs IRR 0.59). A likely explanation for the differing results between the reviews is that we included a higher number of studies that examined different types of programs in the analysis. An additional explanation could be the inclusion of studies with children because injury patterns vary with age [42]. The most obvious difference from other studies was regarding hamstring injuries. The results may be expected as we did not include trials investigating the Nordic Hamstring as a single component exercise, which has been shown to be very effective for preventing hamstring injuries [43]. Moreover, in comparison to Thorborg et al. [14], we included the Bounding Exercise Program [34], which showed very little effect in reducing these injuries.

#### Effectiveness of Injury Prevention Programs on Contact Versus Non-contact Injuries

For the first time, this study investigated the effect of multi-component exercise-based injury prevention programs not only on non-contact injuries but also on contact-related injuries. The point estimate (RR) for contact injuries was 0.70 (95% CI 0.56–0.88). Surprisingly, the estimated risk reduction was higher than for non-contact injuries for which the vast majority of programs are designed. Most programs include strength exercises that mostly focus on core stability. Furthermore, plyometrics (hopping, jumping, and landing) are often part of the programs. They have the potential to improve lower leg strength, functional leg stability, and balance, thus improving the ability to absorb external forces, for example, induced by contact. The 11 + Kids [13] program also includes one exercise specifically on correct falling techniques. The point estimate (RR) for non-contact injuries in the current study was 0.78, in line with a previous study that reported a RR of 0.77 [16].

#### Effectiveness of Injury Prevention Programs Across Sexes and Age Groups

The subgroup analysis showed a point estimate (RR) of 0.70 in male football players. These results mimic the data of the Al Attar et al. study [15]. However, the estimated effect is slightly lower than data reported by Lemes et al. [16] showing a point estimate (RR) of 0.68.

Regarding female individuals, the pooled results showed a point estimate (RR) of 0.82. This result falls within the range of results reported by studies with similar inclusion criteria [15, 16]. However, the meta-analysis with the largest estimated effect [41] included RCTs that used various injury prevention strategies. In addition to physical exercises, they included studies that used braces and education as a method for prevention. Furthermore, they included studies with participants of varying backgrounds and sports (i.e., middle and high school non-footballer athletes). These dissimilarities might have caused these considerable differences. In contrast, small differences compared with other reviews [15, 16] may reflect the diversity of interventions, i.e., the inclusion of single-component exercise-based injury prevention programs.

The subgroup analysis for age groups showed a point estimate (RR) of 0.52 in children, a RR of 0.74 in youth, 0.73 in seniors, and 0.91 in veteran football players. The point estimate in youth and seniors is homogeneous with the current available evidence [14, 41]. The low point estimate found in children may be expected by the fact that there is rarely any prior use of preventative measures at all; therefore, using the program is likely to evoke the biggest benefit. Only one trial [4] assessed the effects of injury prevention programs in veteran football players. The comparably small effect in this population is likely owing to the infrequent application of the program (only once a week) as well as relatively low compliance.

### Factors to Take into Account When Assessing PIs

In the current analysis, we calculated the PIs for the main investigated outcomes. Prediction intervals were wider in comparison to confidence intervals. Based on this evidence, there is a lack of compelling data to affirm the certainty of preventive effects from multi-component exercise-based injury prevention programs. However, for our meta-analysis, we have to take into account that the use of PIs has its shortcomings. IntHout et al. [19] mentioned that they show a wider range compared with CIs when there is any heterogeneity. Our main outcome provided an *I*^2^ = 80.5%, which should be interpreted as high heterogeneity according to the Cochrane Handbook for Systematic Reviews of Interventions [27]. In addition, Riley et al. [40] stated that a PI will be most appropriate when the studies included in the meta-analysis have a low risk of bias. However, the majority of studies in our analysis had a high risk of bias. Therefore, these shortcomings would have affected the use of PIs in our meta-analysis.

### Strengths and Limitations

To the best of our knowledge, this review is the first to analyze the efficacy of multi-component exercise-based injury prevention programs among footballers of all age groups. One strength of this systematic review is that it included multiple analyses. It investigated the risk reduction for the overall number of injuries as well as of body region-specific, contact, and non-contact injuries. Subgroup analyses for age and sex were also performed. Additionally, the PIs for the main outcomes were calculated. A further strength is the large number of participants (22,177), injuries (5080), and exposure hours (1,587,327 h) included in comparison with other reviews [14–16]. Furthermore, we followed best practice by including only randomized trials and cluster-RCTs, using a risk of bias assessment and grading the quality of evidence.

However, this review also has some limitations, mainly that > 50% of the reported effects were based on studies with a very low or low level of evidence. The main outcome variable provided high heterogeneity among the studies (*I*^2^ = 80.5%). The lack of information about compliance with the prevention program in many studies is another limitation of this review. Furthermore, there was missing information on content and compliance with the usual warm-ups/training routines of the control groups. Another limitation is the high risk of bias, especially from the “other bias” domain, with seven studies failing to report the use of an intention-to-treat analysis and of an adjustment for clustering. Finally, two deviations (lack of a compliance analysis and the modification of literature databases) from the original study protocol have to be mentioned as limitations of this review.

### Differences Between the Protocol and Review

Owing to the lack of respective information provided in the studies, a compliance analysis was impossible. We contacted the corresponding authors to provide us with these data, but within the set time of 2 weeks, we only received information on one of the studies. Our planned bibliographic databases for literature identification were modified during the study implementation. Because of the lack of access, we did not search in EMBASE and SPORTDiscus. However, we additionally searched in the originally unplanned database Scopus. In addition, to empower the review, although it was not registered in the protocol, we assessed the quality of evidence using the GRADE approach and calculated the PIs for the main outcomes.

### Recommendations for Future Studies

Based on the data obtained, we recommend future high-quality trials to investigate the efficacy of multi-component exercise-based injury prevention programs. In upcoming studies, data on compliance and the content of the training of the control groups should be included. Adjustment for clustering and more extensive reporting of outcomes should be emphasized. In addition, it appears important to create new injury prevention programs that reflect the development and changes in football training. This should include increasing their attractiveness to promote compliance (also outside of study settings), which appears crucial to reduce injury risk. Currently, a large number of different exercises are included because it is unknown which exercises (or which combination of them) are most effective in general or in relation to specific injuries. Tailoring the exercises would potentially mean fewer injuries and more efficiency.

## Conclusions

This meta-analysis indicated that evidence for the meaningful effects of multi-component exercise-based injury prevention programs in football remains inconclusive at best. This statement is based on PIs that were wider than the frequently employed CIs, with a range from very protective effects to an increased injury risk. In addition, the quality of evidence is a major issue in existing studies. These findings call for future high-quality trials to provide more reliable evidence regarding the efficacy of injury prevention programs in football.

## Supplementary Information

Below is the link to the electronic supplementary material.

Supplementary file1 (PDF 226 KB)

## Declarations

### Availability of data and material

The datasets generated and/or analyzed during the study implementation are available from the corresponding author upon request.
