# Supplementary material for: Efficacy of Multi-Component Exercise-Based Injury Prevention Programs on Injury Risk Among Footballers of All Age Groups: A Systematic Review and Meta-analysis
Source: Sports Med. 2023 Feb 8;53(4):837–48. doi: 10.1007/s40279-022-01797-7 (PMC10036279; doi:10.1007/s40279-022-01797-7)
Supplement: Supplementary file 1 — Supplementary file1 (PDF 226 KB) [file 40279_2022_1797_MOESM1_ESM.pdf]

Abbreviations

IG intervention group, *CG* control group**,** *BEP* bounding exercise program, *RR* risk ratio, I^2 I square, τ^2 Tau square

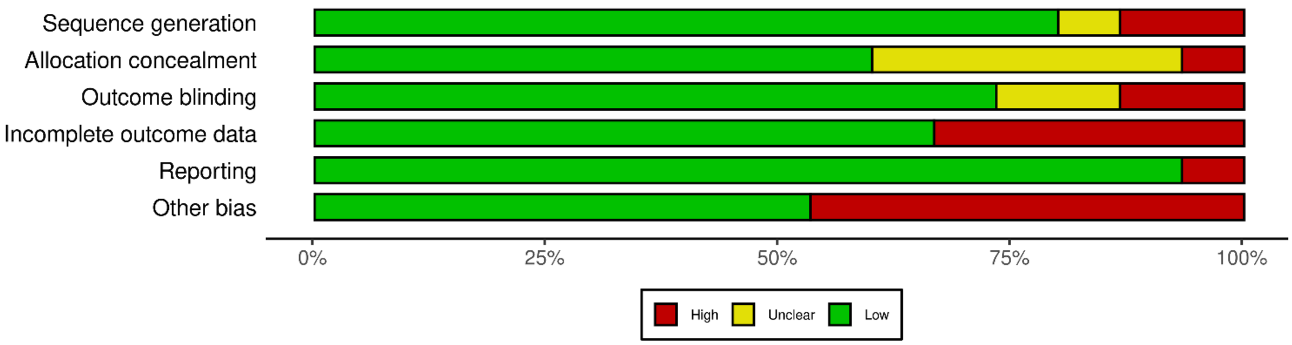

Figure 1. Risk of bias graph: review authors' judgments about each risk of bias item presented as percentages across all included studies

Supplementary Table 1. Methodological quality assessment using the Cochrane Collaboration Risk of Bias Tool

Articles	Sequence generation	Allocation concealment	Participant blinding	Outcome blinding	Incomplete outcome data	Reporting	Other bias
Emery et al. (2010)	Low	High		Low	High	Low	Low
Finch et al. (2016)	High	Low		Low	Low	Low	Low
Gilchrist et al. (2008)	Low	Low		Low	High	Low	High
Hammes et al. (2015)	High	Unclear		Unclear	Low	Low	High
Hilska et al. (2021)	Unclear	Low		Low	Low	Low	Low
Nuhu et al. (2021)	Low	Unclear		Low	Low	Low	Low
Owoeye et al. (2014)	Low	Low		High	Low	Low	High
Rossler et al. (2018)	Low	Low		Low	High	Low	High
Silvers-Granell et al. (2017)	Low	Unclear		Low	High	Low	High
Soligard et al. (2008)	Low	Low		Low	Low	Low	Low
Steffen et al. (2008)	Low	Low		Low	Low	Low	Low
Walden et al. (2012)	Low	Low		Low	Low	Low	Low
Zarei et al. (2020)	Low	Low		Low	Low	Low	High
Van de Beijsterveldt et al. (2012)	Low	Unclear		High	Low	High	High
Van de Hoef et al. (2019)	Low	Unclear		Unclear	High	Low	Low

Supplementary Table 2. Detailed description of injury prevention programs used, frequency and duration.

Study	Description of intervention	Frequency (times/week)	Duration
Emery et al. (2010)	Neuromuscular prevention training: 5 minutes warm-up including aerobic and dynamic stretch components, in addition 10 min of neuromuscular training components (i.e., strength, agility, balance) and a 15-min home-based balance training program (using a 16-inch diameter wobble board)	at least 3x/week	15 minutes + 15 min home-based training
Finch et al. (2016)	Neuromuscular control exercise program (PAFIX): includes plyometric training, balance exercises on (un)stable surfaces, and change of direction tasks[a]	2x/week	Not provided
Gilchrist et al. (2008)	Prevent injury and Enhance Performance (PEP): the program includes: stretching, strengthening, plyometrics, agilities, and avoidance of high-risk positions depicted on a video	3x/week	< 30 minutes
Hammes et al. (2015)	This neuromuscular training program (FIFA® 11+) consists of three parts. The initial part is running exercises at slow speed combined with active stretching and controlled contacts with a partner. The second part consists of six different sets of exercises; these include strength, balance, and jumping exercises, each with three levels of increasing difficulty. Thefinal part is speed running combined with football specific movements with bounding and plant-cut movements	1x/week	20 minutes
Hilska et al. (2021)	Neuromuscular training warm-up: 7 different exercises with focusing on motor skills and movement quality	2-3x/week	20 minutes
Nuhu et al. (2021)	FIFA 11®+[b]	at least 3x/week	20 minutes
Owoeye et al. (2014)	FIFA 11®+[b]	at least 2x/week	20 minutes
Rossler et al. (2018)	11+ Kids: 7 different exercises. 3 exercises focus on (unilateral) dynamic stability of the lower extremities (hopping, jumping and landing), 3 exercises on whole body and trunk strength/stability, and one exercise on falling technique.	at least 2x/week	15-20 minutes
Silvers-Granell et al. (2017)	FIFA 11®+[b]	2-3x/week	20 minutes
Soligard et al. (2008)	FIFA ®11+[b]	2-5x/week	20 minutes
Steffen et al. (2008)	The 11: 10 exercises for core stability, balance, dynamic stabilization and eccentric hamstrings strength	Every session for 15 consecutive sessions, later 1x/week	15 minutes

Walden et al. (2012)	Knäkontroll: exercises focusing on knee control and core stability. 6 exercises: one and two legged knee squat, pelvic lift, bench, lunge, and jump/landing technique. 4 difficulty levels and a pair exercise	2x/week	15 minutes
Zarei et al. (2020)	11+ Kids[c]	At least 2x/week	20 minutes
Van de Beijsterveldt et al. (2012)	The 11[d]	At least 2x/week	10-15 minutes
Van de Hoef et al. (2019)	BEP (bounding exercise program): concentric to eccentric to plyometric exercises and a maintenance program	Not provided	3-5 minutes

[a] information collected from the official web page of the PAFIX program

[b] description of the program is the same as mentioned by Hammes et al.

[c] description of the program is the same as mentioned by Rossler et al.

[d] description of the program is the same as mentioned by Steffen et al.

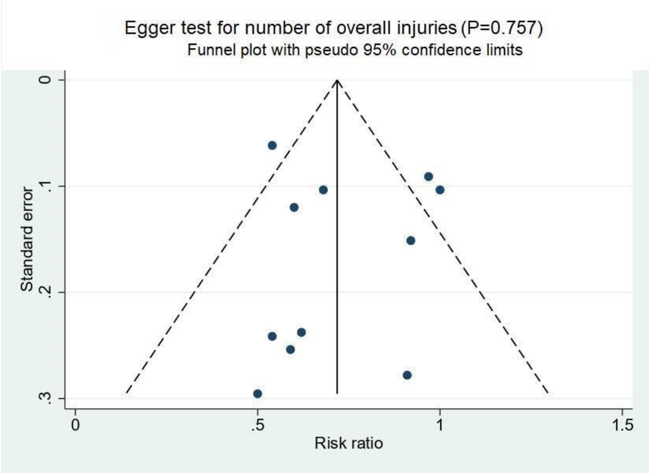

Figure 2. Egger test for the overall number of injuries

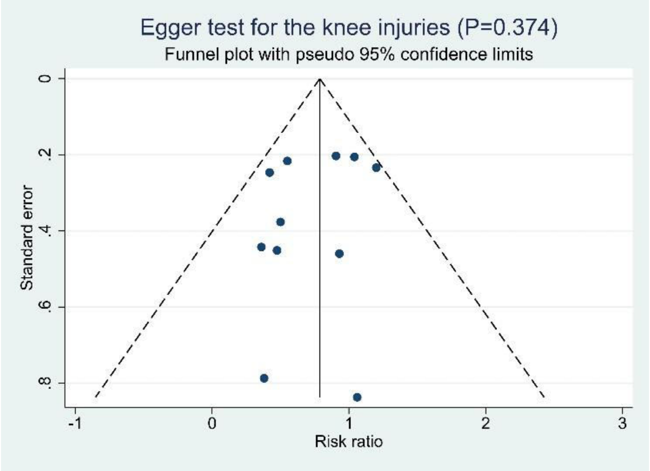

Figure 3. Egger test for the knee injuries

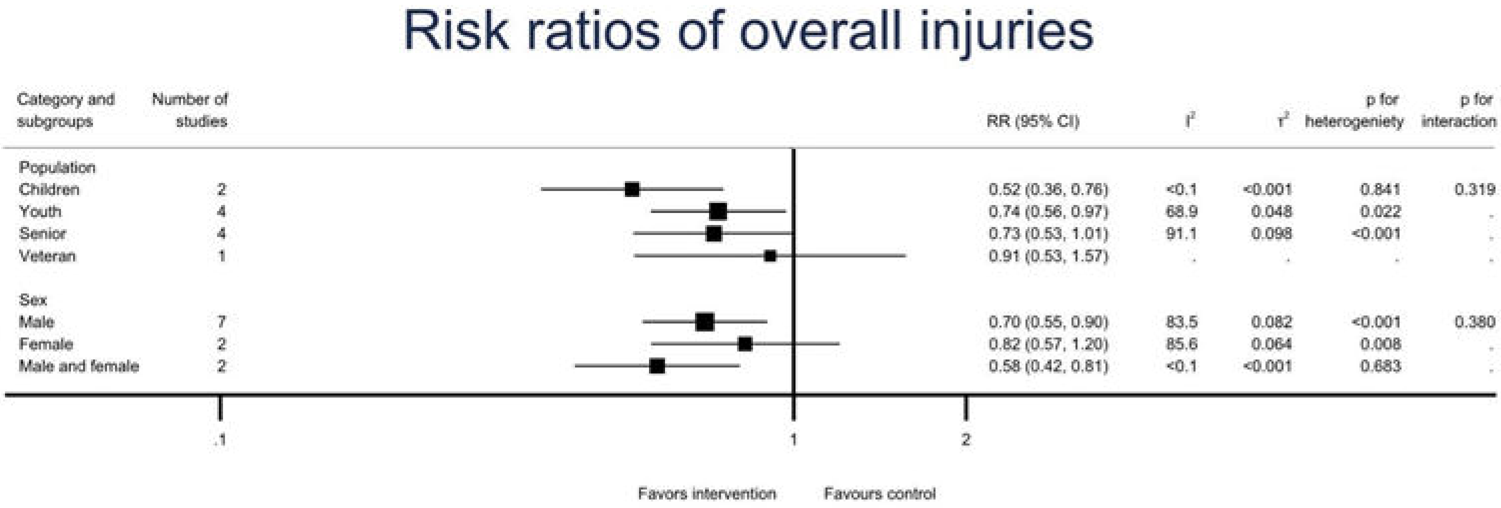

Figure 4. Risk ratios for the overall number of injuries; sub-group analysis according to age-group and sex.
